# Aspirin for Primary Prevention of Cardiovascular Disease and Cancer. A Benefit and Harm Analysis

**DOI:** 10.1371/journal.pone.0127194

**Published:** 2015-07-07

**Authors:** Inge Stegeman, Patrick M. Bossuyt, Tsung Yu, Cynthia Boyd, Milo A. Puhan

**Affiliations:** 1 Clinical Epidemiology, Biostatistics and Bioinformatics, Academic Medical Center, Amsterdam, The Netherlands; 2 Department of Otorhinolaryngology—Head and Neck Surgery, University Medical Center Utrecht, Utrecht, The Netherlands; 3 Department of Epidemiology, Johns Hopkins Bloomberg School of Public Health, Baltimore, Maryland, United States of America; 4 School of Medicine, Johns Hopkins University, Baltimore, Maryland, United States of America; 5 Epidemiology, Biostatistics and Prevention Institute, University of Zurich, Zurich, Switzerland; Virginia Commonwealth University, UNITED STATES

## Abstract

**Background:**

Aspirin is widely used for prevention of cardiovascular disease. In recent years randomized trials also suggested a preventive effect for various types of cancer. We aimed to assess, in a quantitative way, benefits and harms of aspirin for primary prevention of both cardiovascular disease and cancer for a general US population between 40 and 85 years of age.

**Methods:**

We used the Gail/National Cancer Institute approach for assessing benefits and harms. This approach provides a probability that a treatment is more beneficial than harmful and incorporates multiple outcomes, the importance of these outcomes, considers different outcome risks and treats mortality as a competing risk. Our main outcomes were the risks of seven types of cancer, myocardial infarction, ischemic and hemorrhagic stroke and gastrointestinal bleeding. We obtained effect estimates from recent meta-analyses of randomized trials and used baseline risks from the Centers for Disease Control. We conducted four sensitivity analyses to assess the influence of different assumptions about outcome risks and preferences and considered the sampling variation of the effect estimates for aspirin.

**Results:**

The main analysis as well as the sensitivity analyses showed that aspirin has more benefits than harms. In the main analysis, the index (positive if number of prevented events > excess number of harm events over 10 years per 1,000 persons) ranged from 2 (95% CI 0.0 to 11.8; in women age 45 to 54 years) to 8 (95% CI -0.1 to 83.7; in men age 65 to 74 years). In the sensitivity analyses, the index was also positive for all age categories suggesting more benefits than harms.

**Conclusion:**

This study suggests an overall benefit of aspirin for primary prevention of cardiovascular disease and cancer based on population-based data. For individual preventive counseling, additional benefit harm analyses should explore which individuals should or should not take aspirin based on their risk profile for cardiovascular, cancer and gastrointestinal outcomes and based on their outcome preferences. Thereby, risk-stratified and preference-sensitive prevention could become a reality.

## Background

Cardiovascular disease (CVD) and cancer contribute substantially to morbidity and mortality in developed countries. In the USA, cancer incidence rates were 963 per 100,000 persons for 55–59 year olds in 2008, and 2,994 per 100,000 persons for 80–84 year olds. [1,2] Cardiovascular disease accounts for approximately 33% of all deaths in the US. [3–5]Primary prevention of cardiovascular disease and cancer has become an important public health goal. Contemporary preventative measures are often multifaceted and may include life style modification such as increasing physical activity and modifying diet, smoking cessation and drug treatments such as aspirin. [6,7]

While low dose aspirin is widely used in clinical practice for secondary prevention after myocardial infarction and stroke [8–16], low-dose aspirin is increasingly used as a primary prevention measure for cardiovascular disease.[1,8,9] The U.S. Preventive Services Task Force made recommendations about primary prevention for individual patients without cardiovascular disease to prevent stroke and cardiovascular events. [3,5,9] In recent years, the role of aspirin to prevent cancer has also been discussed. Evidence from recent meta-analyses of primary prevention cardiovascular disease trials showed a reduction in cancer incidence, risk for metastasis and cancer specific mortality in aspirin users. These meta-analyses of trials comparing aspirin versus placebo were assessed in order to evaluate the effect of aspirin on major cardiovascular events or stroke in adults[1,8–11,13,14,19]

Despite these benefits, the use of aspirin as a primary prevention measure also has its downsides. Because of the anti-clotting effect, gastrointestinal bleeding is one of the most common complications of long term aspirin use.[1,10] Aspirin inhibits the COX-1 enzyme thereby reducing the synthesis of prostaglandins. As a consequence, the gastric mucosa is less protected from acid-induced damage, and the relative risk of gastric bleeding in aspirin users is 1.62(95% confidence interval (CI), 1.31, 2.00). [1] Hemorrhagic stroke is another rare but potentially severe complication of long-term aspirin use.[5,8,11] In 2005, 23% of 45 to 64 year olds without heart disease in the US took aspirin on a daily or every other day basis.[9] In the category 65 and older this percentage was even higher (41%) despite the fact that it is still unclear whether aspirin for primary prevention provides more benefits than harms. [9,12–16]

Quantitative approaches for developing recommendations can be particularly helpful if the benefit-harm balance is difficult to judge. This is often the case when baseline risks for benefit and harm outcomes vary substantially, when the importance of outcomes vary and when there are competing events such as mortality.[17] Our aim was to assess the benefits and harms of aspirin for primary prevention of both cardiovascular disease and cancer in the general population between 40–85 years of age.

## Methods

### Approach

We specified a population-based decision making context from the perspective of a hypothetical guideline-maker. We wanted to assess the benefits and harms of low dose aspirin compared to no aspirin for primary prevention of cancer and cardiovascular disease. The target population was defined as the adult population without evidence of cardiovascular disease, stroke or cancer, between 40 and 85 years of age. We made separate analyses for men and for women, and allowed estimates of baseline risk of the assessed outcomes to vary with age.

The beneficial outcomes were prevented cases of myocardial infarction, major ischemic stroke and cancer (benefits). The harms were major hemorrhagic stroke and major bleeding, which is primarily gastrointestinal bleeding. The time horizon was 10 years. Death from any cause was considered as a competing risk.

We used the Gail/National Cancer Institute approach for assessing benefits and harms. This approach provides a probability that a treatment is more beneficial than harmful and incorporates multiple outcomes, outcome risks and the importance of outcomes and is therefore more comprehensive in terms of considering multiple data sources than other approaches.[1,18–20] The code can be requested by contacting the corresponding author.

### Data Sources

#### Cardiovascular disease

We used the summary estimates of a random effects meta-analysis for primary prevention of cardiovascular disease by aspirin.[1,8] This study reported a relative risk (RR) of 0.86 (95% CI 0.74;1.00) for myocardial infarction, RR of 0.87 (95% CI 0.73;1.02) for ischemic stroke, RR of 1.35 (95% CI 1.01;1.81) for hemorrhagic stroke, and RR of 1.62 (95% CI, 1.31;2.00) for gastrointestinal bleeding for aspirin compared to placebo (Table 1).

**Table 1:** Data for benefit-harm assessment. Treatment effects and outcome risks.

		**Incidence rates cardiovascular disease and gastrointestinal-bleeding (per 10,000 persons) without aspirin based on surveillance data**							
	**Relative Risk with aspirin (95% CI)**	**Incidence rates in men**				**Incidence rates in women**			
		45–54	55–64	65–74	75–84	45–54	55–64	65–74	75–84
Myocardial infarction	0.86 (0.74;1.00)	40	62	93	140*	12	30	47	82
Major ischemic stroke	0.87 (0.73;1.02)	12	25	56	108	9.0	20	36	75
Major hemorrhagic stroke	1.35 (1.01;1.81)	2.0	4.0	8.0	16	1.0	3.0	5.0	11
Major gastrointestinal bleeding	1.62 (1.31;2.00)	12	25	49	80	6.0	12	23	37
All-cause mortality		50	100	250	670	30	70	160	480
		**Incidence rates cancer (per 10,000 persons)**							
Colorectal cancer	0.63 (0.47;0.85)	9.5	21.5	44.7	65.5	12.5	15	30.5	50.4
Lung cancer	0.96 (0.70;1.32)	9.5	32.7	82.3	114	8.1	24.1	57.3	68.4
Bladder cancer	0.94 (0.64;1.38)	3.3	11.8	32.0	58.0	1.2	3.3	7.9	13.3
Stomach cancer	1.01 (0.54;1.86)	1.0	3.7	7.8	11.7	0.8	1.6	3.4	5.9
Pancreas cancer	1.07 (0.59;1.94)	2.0	5.6	11.9	17.4	1.3	3.8	8.7	14.7
Oesophagus cancer	0.76 (0.38;1.53)	1.5	4.4	7.7	9.8	0.3	0.8	1.7	2.5
Prostate cancer (men)Breast cancer (women)	0.87 (0.69;1.10) 0.90 (0.26;3.07)	20.3	92.6	182.7	153.8	12.8	16.4	19.8	15.6
Cancer overall men/women	0.75 (0.59;0.94) 0.77 (0.63;0.93)	86.2	219.1	500.7	620	100.9	180.9	299	373.4
	**Incidence rates (per 10,000 persons) without aspirin. For sensitivity analyses 4, with RCT data. Mean ages 53–64 years.**								
	**Men**					**Women**			
Myocardial infarction	56					10			
Major ischemic stroke	21					11			
Major hemorrhagic stroke	3.0					2.0			
Major gastrointestinal bleeding	9.0					5.0			
All-cause mortality	79					34			

* No reliable data was available from the Atherosclerosis Risk in Communities Study for men in age category 75–84 years. To estimate the incidence rate we assumed a 50 percent increase from age category 65–74 years based on similar increases in incidences in age category 65–74 to 75–84 in the Framingham Heart Study and the Cardiovascular Health Study.

#### Cancers

For colorectal, lung, esophageal, pancreatic, stomach, prostate, breast and bladder cancer, we used the results from a large trial on the effects of aspirin on cancer incidence.[1,13,16,21–25]. This study reported a hazard ratio (HR) for colorectal cancer of 0.63 (95% CI 0.47;0.85), a HR for lung cancer of 0.96 (95% CI 0.70;1.32), a HR for esophageal cancer of 0.76 (95% CI 0.38;1.53), a HR for pancreatic cancer of 1.07 (95% CI 0.59;1.94), a HR for stomach cancer of 1.01 (95% CI 0.54;1.86), a HR for bladder cancer of 0.94 (95% CI 0.64;1.38), a HR for prostate cancer of 0.87 (95% CI 0.69;1.10) and a HR for breast cancer of 0.90 (95% CI 0.26;3.07). (Table 1)

### Outcome risks

#### Baseline Risks Cardiovascular disease

Table 1 shows the estimated incidence rates without aspirin for cancer, cardiovascular disease and gastrointestinal-bleeding. We used data from the Atherosclerosis Risk in Communities Study, a prospective epidemiologic study conducted in four U.S. communities, to obtain baseline incidence rates of myocardial infarction and stroke without treatment. [5,9,11,26] These data are used by the National Heart, Lung & Blood Institute to report trends on the incidence of cardiovascular disease. No reliable data were available for men in the age category 75–84 years. To estimate the incidence rate we assumed a 50 percent increase from age category 65–74 years based on similar increases in incidences in age category 65–74 to 75–84 years in the Framingham Heart Study and the Cardiovascular Health Study. [27,28]

Since we were unable to find appropriate population-based estimates of gastrointestinal bleeding in the US population we relied, similar to what was done by the U.S. Preventive Services Task Force in their aspirin guideline, on estimates from the General Practice Research Database, a database from the United Kingdom, and the Base de Datos para la Investigacion Farmacoepidemioloica en Atecion Primaria from Spain. This report provided information about the prevalence of risk factors for severe GI bleeding (age, sex, prior GI history) and incidence rates for severe GI bleeding for several age and sex categories. For our four age categories we calculated the incidence of severe GI bleeding. [29,30] To estimate the all-causes risk of death we relied on estimates from the Center for Disease Control. [31] We made no correction for double counting of causes of death.

#### Baseline risks cancer

Table 1 shows the estimates of the incidence rates without aspirin for the types of cancer in our study based on data from the Centers for Disease Control. [12–16,25]

### Assumptions

To perform the benefit-harm analyses a number of additional assumptions had to be made. First of all, we assumed no heterogeneity in the relative effects of aspirin for either benefits or harms. This assumption is based on the results of the meta-analyses by Berger, Flossmann and Rothwell. [1,10,13,18,21] We additionally assumed that the relative effects of aspirin on benefits and harms did not vary between men and women[1,8,25] and that they were constant over time. We assumed that all cases of myocardial infarction were all of the same severity, that all strokes were severe, and that all episodes of major bleeding were major upper or lower gastrointestinal bleeds. We assumed that all cancers were equally severe.

### Statistical analysis

#### Summary index for benefits and harms

The Gail/NCI approach^19^ considers multiple patient-important outcomes of a medical intervention and provides profile-specific estimates of the benefit-harm balance. In other words, the Gail/NCI approach provides a quantitative benefit-harm comparison that indicates whether treatment compared to no treatment will lower or increase the incidence of patient-important outcomes, over a specified period of time for patients of a specific age and sex. An index >0 means that the benefits outweigh the harms; an index <0 indicate more harms than benefits. The probabilities refers to the probability that a specific index (e.g. for men with 55–64 years of age) is positive.

Necessary calculations are described below. In a first step we calculated the number of events for each of the outcomes described above, expressed per 1,000 subjects over 10 years, using mortality and incidence rates. We used the following equation

Nx,p=1,000×{(Ix/(Ix+M)}×[1−exp{−10(Ix+M)}] (1)

where Nx,p is the number of events for a specific outcome (x) per 1000 subjects over 10 years in subjects without aspirin, Ix is the incidence of the event of interest, and M is the all-cause mortality.

We then calculated the corresponding number of events with aspirin, again for each of the outcomes, also expressed per 1,000 subjects over 10 years. We used

Nx,t=1,000×{(Rx×Ix/(Rx×Ix+M)}×[1−exp{−10(Rx×Ix+M)}] (2)

where Rx represents the treatment effect of aspirin on the respective outcome.

Then we calculated the Nx, the difference in number of events per 1,000 over 10 years based on Eq (1) and (2).:

Nx=Nx,p−Nx,t (3)

For benefits, Nx is positive: it expresses the number of events prevented by aspirin. For harms, Nx is negative: it expresses the number of additional events induced by the use of aspirin, per 1,000 subjects over 10 years.

The final benefit-harm index is based on summing of all events:

Index=∑Wx*Nx (4)

where W_1_ expresses the relative weight attached to the first outcome, W_2_ to the second, and so for all outcomes considered. In the main analysis we used equal weights for all outcomes. The index is an absolute measure of the benefit-harm balance.

To take into consideration the sampling variability for the treatment effect estimates we used a Bayesian approach and simulations to estimate the posterior probability that the index is positive[19]. A probability of >50% means that that the benefits are greater than the harms (index positive).

### Main and sensitivity analyses

In the main analysis we used a weight of 1.0 for MI, stroke and gastrointestinal bleeding. Since we had multiple cancer outcomes that may not occur independently from one another we used a weight of 0.5 instead of 1.0 for cancers in order not to overestimate the benefits of aspirin. We conducted four sensitivity analyses to consider variations in the data sources for outcome risks and importance of outcomes. In the first sensitivity analysis we used different weights in Eq (4). Weights were assigned according to the estimated five year survival as an indicator of the importance of the outcomes (Table 2). [1,13,16,22–25] In the absence of five year data we conservatively took available three year survival data for GI bleeding. Weights of 1.0, 0.5 or 0.1 were assigned to, 0% to 50%, 50% to 80% and 80% or higher five year survival rates, respectively. Again, we divided the weights for cancers outcomes by two because of multiplicity and in order not to overestimate the benefits of aspirin.

**Table 2:** Weights used in the first sensitivity analysis, based on 5-year survival.

	Male	Female	Weight
Colorectal cancer(22,25)	65%	64%	0.25
Lung cancer(24,25)	14%	19%	0.5
Bladder cancer(24,25)	80%	73%	0.25
Stomach cancer(23,25)	24%	28%	0.5
Pancreas cancer(5,25)	5.4%	6.0%	0.5
Oesophagus cancer(9,25)	18%	17%	0.5
Prostate/Breast Cancer (25,28)	99%	89%	0.05
Myocardial infarction(13,22)	79%	79%	0.25
Haemorrhagic stroke(15,24)	42%	42%	0.5
Ischemic stroke(1,24)	42%	42%	0.5
Gastrointestinal bleeding(23,34)	63%	63%	0.25

In the second sensitivity analysis we used treatment effects for overall cancer risk, because overall incidence rates of cancer are higher than the sum of the separate cancers. We used a meta-analysis reported by Rothwell.[9,25,26] The summary estimate for overall cancer was an OR of 0.75 (95% CI 0.59;0.94) for women and 0.77 (95% CI 0.63;0.93) for men. In the third sensitivity analysis we used again treatment effects for overall cancer risk and weights of 0.5 for MI, 1.0 for stroke, 0.5 for gastrointestinal bleeding and 1.0 for overall cancer.

In the fourth sensitivity analysis we used data from RCT’s for the incidence rate estimates, because outcome risks from control groups in trials are often used to calculate the absolute effect of treatments. However, we considered these control group outcome risks (Table 1) only a sensitivity analysis, since RCT participants are often selected and may not represent the outcome risks in a general population. As an example, most RCTs excluded people with prior gastrointestinal bleeds. Data were not available for the four age categories. Mean participant age in the RCT considered ranged from 53 to 65, which is comparable to the age category 55–64.

## Results

Table 3 shows the number of expected events over 10 years for 1,000 men and women, with and without aspirin. For example, in men between 55 and 64 years the expected cases of myocardial infarctions in those taking aspirin would be 49 per 1,000 over 10 years. In the same age group we expect 57 myocardial infarctions in 1,000 men not taking aspirin, also over 10 years. In Table 4 the numbers of expected events with and without aspirin are shown as well as the results of the sensitivity analyses. In the main analysis, the index (positive if prevented benefit events > excess harm events over 10 years per 1,000 persons) ranged from 2 (95% CI (-0.1;22.2)) in men with age 45 to 54 years to 8 (95% CI -0.1;83.7)) in men with age 65 to 74 years. In the sensitivity analyses, the index was also positive for all age categories suggesting more benefits than harms but again the 95% CI were relatively wide. Probabilities that the index is positive were all above 95%.

**Table 3:** Expected number of events without and with aspirin prevention in men and women. *

	**Number of expected events over 10 years per 1,000 men**							
**Men**								
Age (years)	45–54		55–64		65–74		75–84	
Aspirin (no or yes)	No	Yes	No	Yes	No	Yes	No	Yes
Myocardial infarction	38	33	57	49	79	68	96	83
Cancer overall	81	61	188	144	352	279	295	233
Colorectal Cancer	9	6	20	13	39	25	46	30
Lung Cancer	9	9	31	29	70	67	79	76
Bladder Cancer	3	3	11	10	28	26	41	39
Stomach Cancer	1	1	4	4	7	7	8	9
Pancreas Cancer	2	2	5	6	10	11	13	13
Esophageal Cancer	1	1	4	3	7	5	7	5
Prostate Cancer	20	17	84	74	148	130	105	92
Major ischemic stroke	12	10	24	20	48	42	75	66
Major haemorrhagic stroke	2	3	4	5	7	10	12	16
Major gastrointestinal bleeding	12	19	24	38	42	68	56	89
**Women**	**Number of expected events over 10 years per 1,000 women**							
Age (years)	45–54		55–64		65–74		75–84	
Aspirin (no or yes)	No	Yes	No	Yes	No	Yes	No	yes
Myocardial infarction	12	10	29	25	42	37	63	54
Cancer overall	95	74	160	126	240	191	251	201
Colorectal Cancer	12	8	14	9	28	18	39	25
Lung Cancer	8	8	23	22	51	49	53	51
Bladder Cancer	1	1	3	3	7	7	10	10
Stomach Cancer	1	1	2	2	3	3	5	5
Pancreas Cancer	1	1	4	4	8	9	12	12
Esophageal Cancer	0	0	1	1	2	1	2	2
Breast cancer	13	11	16	14	18	16	12	11
Major ischemic stroke	9	8	19	17	33	29	58	50
Major haemorrhagic stroke	1	1	3	4	5	6	9	12
Major gastrointestinal bleeding	6	10	12	19	21	34	29	46

*All-cause mortality is considered as a competing risk.

**Table 4:** Benefit-harms comparison index for primary prevention of cardiovascular events and cancer with low dose aspirin over 10 years per 1,000 persons (95% CI and probability that index is positive based on recalculating the index in simulations).

	Men				Women			
	45–54 (95%CI; probability)	55–64(95%CI; probability)	65–74(95%CI; probability)	75–84(95%CI; probability)	45–54(95%CI; probability)	55–64(95%CI; probability)	65–74(95%CI; probability)	75–84(95%CI; probability)
**Main analysis** ^**1**^ **Type specific cancer rateSame weights for all outcomes**	2 (-0.1, 22.2; 97%)	5 (0.1, 47.9; 98%)	8 (-0.1, 83.7; 97%)	3 (-0.2, 97.7; 97%)	2 (0.0, 11.8; 98%)	2 (0.1, 24.0; 98%)	3 (-0.2, 41.0; 97%)	4 (0.1, 57.3; 98%)
**Sensitivity analysis 1; Type specific cancer rateWeights based on five year survival**	1 (0.0, 12.7; 98%)	2 (0.1, 26.1; 98%)	3 (-0.1, 47.5; 97%)	3 (-0.2, 61.1; 97%)	1 (0.0, 7.2; 97%)	2 (0.0, 15.9; 97%)	3 (0.0, 28.5; 98%)	4 (0.1, 40.9; 98%)
**Sensitivity analysis 2; Overall cancers ratesSame weights for all outcomes**	18 (0.1, 36.6; 98%)	38 (0.2, 72.6; 98%)	62 (0.3, 120.9; 98%)	55 (0.4, 133.3; 98%)	20 (0.1, 30.8; 98%)	33 (0.2, 54.9; 98%)	45 (0.2, 81.7; 98%)	46 (0.3, 97.5; 98%)
**Sensitivity analysis 3; Overall cancer ratesWeights based on five year survival**	19 (0.1, 30.2; 98%)	42 (0.2, 62,3; 98%)	69 (0.3, 105.6; 98%)	66 (0.4, 114.2; 98%)	21 (0.1, 28.5; 98%)	34 (0.2, 49.8; 98%)	48 (0.2, 73.6; 98%)	50 (0.3, 85.8; 98%)
**Sensitivity analysis 4; Outcome risks based on control groups from RCTsSame weights for all outcomes**	14 (0.0, 22.4; 97%)				3 (0.0, 10.4; 98%)			

^1^Positive values = aspirin beneficial; Negative values = aspirin harmful

## Discussion

In this study we have assessed profile-specific estimates for the benefits and harms of using aspirin for primary prevention in cardiovascular disease and cancer. Several methods for benefit-harm analyses exist, we chose to use the Gail/NCI approach. The Gail/NCI approach considers all key elements to estimate a benefit-harm balance; it considers scientific evidence on treatment effects, patient characteristics with associated risks of outcomes and considers the preferences for these outcomes. The outcome, an index with a probability estimate is likely to be useful in decision making. Our analyses suggest, irrespective of how much weight we assigned to the different outcomes, that in the general US population between 40 and 85 years of age the overall benefits of the use of aspirin as a primary prevention tool when considering outcomes of cancer, cardiovascular risk and gastrointestinal bleeding, are greater than the harms. Whether these results should lead to public health action or revision of some guideline recommendations depends on the context and on cost issues. In our view, the benefit harm index—and probabilities for net benefit associated with it—are but one component informing decisions; other factors also require consideration.

Some limitations need to be addressed. Assumptions have to be made in benefit-harm analyses. In quantitative benefit-harm analyses the importance of the selection of data and sensitivity analyses cannot be stressed too much. The analyses consistently show a consistent overall benefit of aspirin but there are differences in the magnitude of the index across the main and sensitivity analyses. For example, the choice of weights for the outcomes is often arbitrary. However, quantitative approaches like the Gail/NCI approach contribute to understanding how much different weights change the overall benefit harm balance. Furthermore, sensitivity analyses give an opportunity for exploring the impact of characteristics such as age, gender or other prognostic indicators. The use of transparent quantitative approaches where the data sources are clearly presented offers an opportunity to illustrate how different opinions about the appropriate selection of data sources impact on the overall benefit-harm balance.[17,20]

To our knowledge, our study is the first to assess the combined effect of aspirin for primary prevention of both cardiovascular disease and cancer in a single quantitative benefit-harm analysis. The effect of aspirin on cardiovascular disease has been studied for decades whereas the preventable effect in cancer has been studied more recently. Meta-analyses have been conducted for several types of cancer, showing a protective effect for aspirin in the development of cancer.[1,2,4,13,15]^,^ Berger et al. conducted a meta-analysis to investigate the effect of aspirin on cardiovascular disease and harms and concluded that there was only a modest support for benefit of aspirin in patients without cardiovascular disease.[1,26,32] A meta-analysis of Seshasai et al. [33,34] showed that aspirin in primary prevention does decrease the risk of MI, but that aspirin does not lead to lower cancer mortality and increases the risk of bleeding. Our data show that cancer incidence is modestly decreased and that the risks of bleeding are smaller than the benefits of taking aspirin. By conducting a quantitative benefit-harm analysis a more precise estimate of the benefits and harms could be made. By including cancer and more precisely several subtypes of cancer into our analyses we provide a more comprehensive estimate of the benefit to harm ratio, instead of only including cardiovascular disease.

In 2005, 23% of 45 to 64 year olds without heart disease in the US took aspirin on a daily or every other day basis. Recommendations on aspirin do not entirely agree in major guidelines For example, the American College of Chest Physicians (ACCP) (2012) suggest the use of aspirin for persons over age 50 without symptomatic cardiovascular disease, while the US Preventive Services Task Force encourages the use of aspirin in selected populations only (men 45–79 for the prevention of MI and women 55–79 for the prevention of stroke) who are not at elevated risk for bleeding. [5] Except for the US Preventive Services Task Force guideline, the guidelines only take the benefits of aspirin explicitly into account while the consideration of harms, is not made entirely clear. The inconsistencies in recommendations and how to consider both benefits and harms of aspirin may be a consequence of not using formal methods for quantitative benefit-harm analyses. Putting the preventive effects of aspirin as estimated from RCTs into the context of various outcome risks and importance of outcomes is a complex task but analyses such as ours may help. While we do not want to make recommendations about aspirin, our analyses show that it is possible to deal with the multidimensional issue of aspirin for primary prevention in a transparent way. The use of quantitative benefit-harm analysis, where the data used are explicitly described and assumptions are tested in sensitivity analyses, could lead to a better understanding if and for whom a preventive intervention should be recommended or not. It is relatively straightforward to repeat the analyses using different treatment effects, outcome risks and preferences (importance of outcomes) depending on the decision making context. Thereby, the impact of using different data and assumptions on the benefit-harm balance can be tested. These sensitivity analyses are useful because they inform developing the strength of a recommendation. If the sensitivity analyses show similar results favoring a preventive intervention a guideline panel may issue a strong recommendation. In turn, if the sensitivity analyses show different results, the recommendation may be weak for or against the intervention.

We must consider that our analyses were made from a population perspective. For some individual patients, with individual circumstances such as increased risk for gastrointestinal bleeding, aspirin may be associated with more harms than benefits. The risk for gastrointestinal bleeding is increased by older age, prior gastrointestinal events, the use of non-steroidal anti-inflammatory agents and in males.[29] The US preventive Services Task Force recommended that decisions on individual patients should be made on the basis of these risk factors. The Task Force recommends the use of aspirin as a preventive tool when the potential benefit of a reduction in myocardial infarctions outweighs the potential harm of an increase in bleeding. Although the guideline refers to the use of risk prediction models[28], it does not give explicit guidance on how to estimate the balance between harms and benefits for individuals. [5]

This study suggests an overall benefit of aspirin for primary prevention of cardiovascular disease and cancer based on population-based data. For preventive counseling, additional benefit-harm analyses should explore which individuals should or should not take aspirin based on their risk profile for cardiovascular, cancer and gastrointestinal outcomes and based on their outcome preferences. Thereby, risk-stratified and preference-sensitive prevention could become a reality.
